# Pharmacological Inactivation of Medial Prefrontal Cortex Does Not Support Dichotomous “Go/Stop” Roles for Dorsal and Ventral Subdivisions in Natural Reward Seeking in Rats

**DOI:** 10.1523/ENEURO.0246-20.2020

**Published:** 2020-07-09

**Authors:** Rosalind S.E. Carney

## Abstract

**Highlighted Research Paper:** Differential Effects of Dorsal and Ventral Medial Prefrontal Cortex Inactivation during Natural Reward Seeking, Extinction, and Cue-Induced Reinstatement. Jessica P. Caballero, Garrett B. Scarpa, Luke Remage-Healey, David E. Moorman.

Brain regions that evolved to respond to natural rewards, such as food, are also stimulated by drugs of abuse resulting in addiction and relapse, conditions which are uniquely human (Kelley and Berridge, 2002). Identification of the neural substrates of addiction and relapse in humans is limited by feasible experimental approaches and confounding variables such as polysubstance drug use, unreliable reporting of drug use, interindividual variation in the amount, frequency, type of drug use, and periods of abstinence. Non-invasive imaging studies have shown heightened neural activity in the prefrontal cortex (PFC) of human subjects viewing drug-related cues (videos or images) or in response to intravenous drug administration after a brief period of abstinence (Garavan et al., 2000; Kufahl et al., 2005, 2008; Heinz et al., 2007). It is hard to think of a brain region with more complexity and contention in anatomical and functional homology and nomenclature use between rodents and humans than the PFC (Laubach et al., 2018). In rodents, the medial PFC (mPFC), which is part of the mesocorticolimbic system, contains dorsal and ventral subdivisions (dmPFC and vmPFC) that have been analyzed separately for their respective roles in fear learning, drug seeking, and natural reward seeking. Within the dmPFC, the prelimbic cortex (PL) is thought to promote behavioral responses whereas the infralimbic cortex (IL), located within the vmPFC, is thought to suppress behavioral responses (Peters et al., 2009; Gass and Chandler, 2013; Gourley and Taylor, 2016). These opposing behavioral responses form the basis of the “Go(PL)/Stop(IL)” model for mPFC executive function, which would support differential targeting of the dmPFC and vmPFC for potential therapeutic options for addiction and relapse. In a simplified scenario based on the Go/Stop model, inhibiting dmPFC activity may mitigate the likelihood that maladaptive behaviors become habitual, and both inhibiting dmPFC activity and enhancing vmPFC activity may decrease susceptibly to relapse. The Go/Stop model appears to be more consistently valid for fear conditioning and for some aspects of heroin and cocaine drug seeking in rodents (McFarland and Kalivas, 2001; McLaughlin and See, 2003; Fuchs et al., 2005; LaLumiere and Kalivas, 2008; Peters et al., 2008; Muller Ewald and LaLumiere, 2018). Other addiction studies have shown that the dichotomous Go/Stop model of mPFC executive function is not universally applicable across species or experimental contexts (such as the timing of experimental manipulation relative to extinction) in relation to reward-seeking behaviors (McFarland et al., 2003; Jonkman et al., 2009; Bossert et al., 2011; Chen et al., 2013; Willcocks and McNally, 2013; Martín-García et al., 2014; Moorman and Aston-Jones, 2015; Moorman et al., 2015; McGlinchey et al., 2016; Gutman et al., 2017). As many prior studies have focused on drug of abuse, in their *eNeuro* publication, Caballero and colleagues used pharmacological inactivation to investigate further the separate roles for the dmPFC and vmPFC in motivated behavioral responses to sucrose, a natural reward.

Rodents can be trained to self-administer drugs or natural rewards via defined operant behavioral responses that serve as a measure of motivation with respect to reward-seeking behavior. Drug exposure positively reinforces the rodents’ behavior such that further efforts to self-administer are a direct consequence of the stimulating effects of the drug on the brain’s reward system (Lynch et al., 2010). Manipulation of this behavioral paradigm to include extinction and cue-induced reinstatement can produce a measure of drug relapse (Epstein et al., 2006). To determine the separate roles of the dmPFC and vmPFC in different aspects of reward-seeking behaviors, adults rats were bilaterally implanted with cannulae to administer artificial cerebrospinal fluid (aCSF; control) or a mixture of baclofen and muscimol (BM; GABA-A and GABA-B receptor agonists dissolved in aCSF) immediately before experimentation. The experimental approaches and main findings of Caballero and colleagues are described separately for motivation, extinction, and cue-induced reinstatement, which were examined in distinct cohorts of rats to eliminate confounding factors.

## Motivation

**Figure 1.**
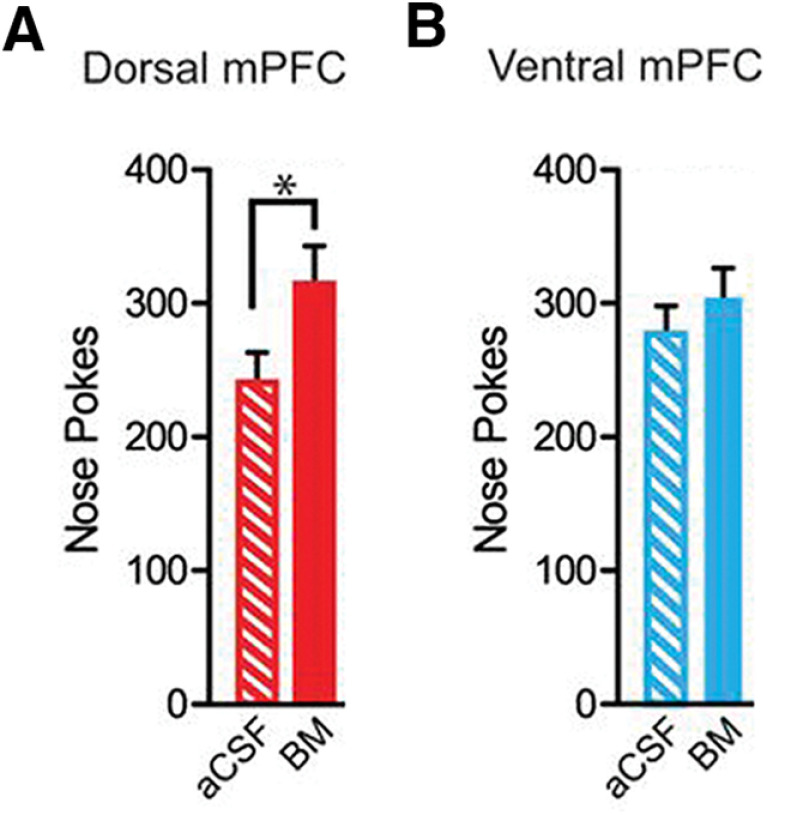
Bilateral inactivation of dmPFC, but not vmPFC, reduces the total number of nose pokes. ***A***, There was a significant increase in total number of nose pokes and when the dorsal mPFC was bilaterally inactivated. ***B***, In contrast, vmPFC inactivation did not affect nose poking. aCSF, control infusion; BM, baclofen and muscimol; *p <* *0.05 (Adapted from Figure 1 in Caballero *et al*., 2019).

Rats were trained on an operant fixed ratio 1 (FR1) schedule in which each response in the active nose poke hole was rewarded with a single delivery of the sucrose reward in a well beneath the nose poke hole. A poke in the inactive hole did not elicit a reward. Temporal pairing of a discrete environmental cue, an auditory tone, with the reward-linked operant response was used to facilitate associative learning of cue–reward memories. Therefore, the rats learned to expect to receive the reward when they heard the tone following a nose poke in the active hole. The rats were trained until a consistent, rapid operant response to the reward was achieved. Subsequent to BM administration, the number of nose pokes and well entries was recorded when either the dmPFC or vmPFC was inactivated, compared with the control (aCSF-administered) group in which these structures functioned normally. An increase in the number of nose pokes or well entries is positively correlated with heightened motivation for the reward. The authors found that inactivation of the dmPFC, but not the vmPFC, resulted in an increased number of nose pokes (Fig. 1). Similar findings were observed for well entries. These observations indicate that inactivation of the mPFC results in an increase in motivational behavior.

## Extinction

**Figure 2.**
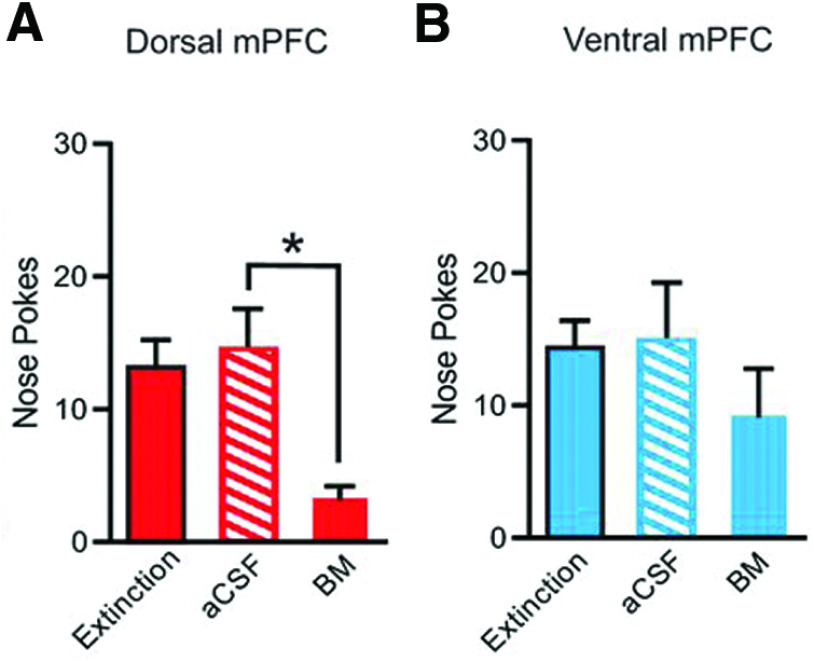
Bilateral inactivation of dmPFC, but not vmPFC, reduces the total number of nose pokes during late extinction. Rats were trained on FR1; bilateral infusions were only administered during late extinction. ***A***, Inactivation of dmPFC during late extinction decreased nose pokes. ***B***, There was no effect of vmPFC inactivation for number of nose pokes during late extinction. The extinction condition refers to behavior on the last day of extinction before testing with aCSF or BM occurred. aCSF, control infusion; BM, baclofen and muscimol; *p <* *0.05 (Adapted from Figure 2 in Caballero *et al*., 2019).

The rats were trained on the FR1 schedule as for the motivation paradigm. Over multiple (early and late) extinction sessions, the strength of cue–reward memories weakened as neither the reward nor the auditory tone followed a nose poke in the active hole. The inactive nose poke was inaccessible during all extinction sessions. Memories are more stably encoded in late extinction compared with early extinction. Bilateral infusion of aCSF or BM only occurred during extinction sessions and not during training to eliminate potential confounds and to limit dmPFC/vmPFC inactivation to the extinction paradigm only. Inactivation of the dmPFC resulted in a lower total number of nose pokes during late extinction compared with the aCSF group (Fig. 2). Inactivation of the vmPFC led to a small decrease during late extinction in the total number of nose pokes that was not statistically significant from those emitted during the last extinction session immediately before testing (Fig. 2). During late extinction, well entries were reduced by inactivation of either the dmPFC or the vmPFC.

## Cue-Induced Reinstatement

**Figure 3.**
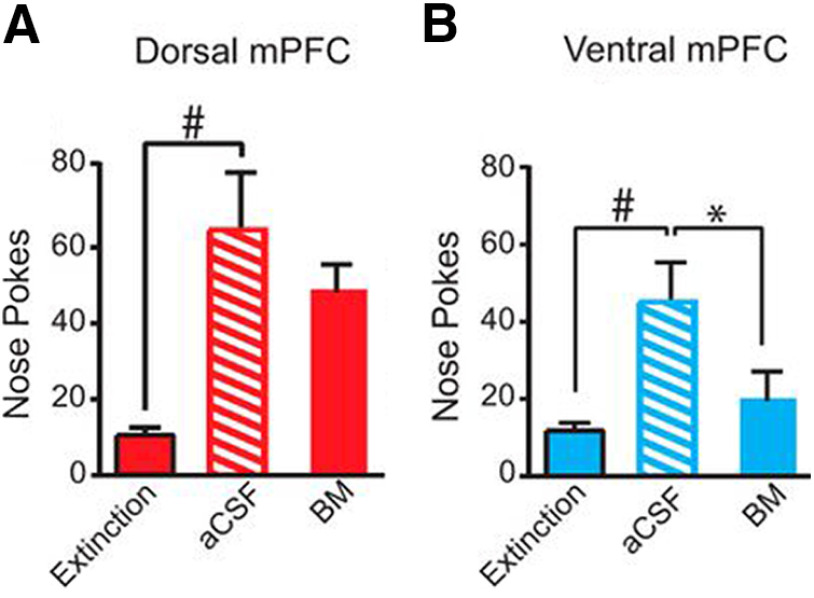
Bilateral inactivation of vmPFC reduces the total number of nose pokes during reinstatement, whereas no effect was found for dmPFC bilateral inactivation. Reinstatement rats were trained on FR1; bilateral infusions were only administered during reinstatement. There was a significant increase in nose pokes on aCSF reinstatement infusion day compared with last day of extinction. ***A***, Bilateral inactivation of dmPFC did not significantly affect nose pokes. ***B***, Bilateral vmPFC inactivation significantly decreased total number of nose pokes. aCSF, control infusion; BM, baclofen and muscimol; *p <* *0.05 (Adapted from Figure 3 in Caballero *et al*., 2019).

Rats were trained on the FR1 and extinction paradigms. When the conditioned operant response was consistently and satisfactorily extinguished in a separate cohort of rats, cue-induced reinstatement was used to reintroduce the environmental cue without reward delivery. In this case, a renewed interest in the (unavailable) reward, stimulated by the return of the auditory cue, served as a measure of addiction relapse. Bilateral infusion of aCSF or BM only occurred immediately before cue-induced reinstatement testing and was not performed during FR1 or extinction. Inactivation of the dmPFC did not significantly affect the total number of nose pokes (Fig. 3). In contrast, vmPFC inactivation lead to a significant decrease in the total number of nose pokes (Fig. 3). No effect of dmPFC or vmPFC inactivation was observed for well entries compared with aCSF-administered control rats.

Nose pokes and well entries are only two highlighted variables that were examined during motivation, extinction, and cue-induced reinstatement experiments. Collectively, the findings of Caballero and colleagues exhibit the following similarities and differences with respect to the Go/Stop model of dmPFC and vmPFC roles in reward-seeking behavior:

- Motivation: the Go/Stop model predicts that dmPFC and vmPFC inactivation would decrease and increase sucrose seeking respectively during FR1 self-administration. Caballero and colleagues found that inactivation of the dmPFC increased sucrose seeking while vmPFC had no effect on FR1 self-administration.
- Extinction: the Go/Stop model predicts that dmPFC inactivation would not affect extinction, whereas vmPFC inactivation would increase sucrose seeking during extinction. Caballero and colleagues found that during extinction, inactivation of the dmPFC or vmPFC both decreased reward seeking.
- Cue-induced reinstatement: the Go/Stop model predicts that vmPFC inactivation, but not dmPFC inactivation, would result in a relapse phenotype. Caballero and colleagues found that dmPFC inactivation did not affect cue-induced reinstatement, while vmPFC inactivation resulted in reduced sucrose seeking. Caballero and colleagues found that reward seeking during cue-induced reinstatement was reduced with vmPFC, but not dmPFC, inactivation.

This *eNeuro* publication is an advance in the field because it provides further support that the dichotomous Go/Stop role for the PL and IL does not universally apply to motivated behaviors. Moreover, the results reveal further insight into the contribution of the dmPFC and vmPFC in reward seeking and relapse behavior in response to a natural reward. Both drugs of abuse and strongly palatable food rewards alter neuronal plasticity in the reward circuit (Russo et al., 2010; Guegan et al., 2013). Therefore, it is important to dissect differential contributions of the dmPFC and vmPFC with the consideration of structural alterations that arise from the pathological damage manifested by addictive substances. For example, marked changes in functional connectivity were observed between separate baseline (preexposure) and short-term periods of abstinence from cocaine exposure in rats (Orsini et al., 2018). For human relevance, it is therefore crucial to distinguish postaddiction alterations of neural substrates from preexisting or comorbid anomalies that increase susceptibility to addiction. As the brain signaling pathways that mediate addiction behaviors during initial and chronic drug exposure may differ (Lynch et al., 2010), the reality of manipulating mPFC function to combat substance abuse is exceptionally complex. As the mPFC is involved in multiple brain processes, therapeutic treatments that target mPFC activity may be clinically relevant to other neurological disorders such as anxiety. However, the complexity of the mPFC in terms of anatomical structure and interactions with other brains regions challenge a broadly applicable therapeutic manipulation of mPFC output (Xu et al., 2019).

Caballero and colleagues’ *eNeuro* publication highlights that different experimental variables and contexts create both caveats and increased potential to further knowledge of the mPFC contribution to addiction and relapse. As addiction is a human disorder, the translatability of results from rodent models is somewhat limited. Fortunately, continuous improvement in experimental design provides new avenues to understand the adverse effects of substance abuse in a context more relevant to humans. A recent experimental advance includes the incorporation of a social interaction component of reward behavior in rodents (Venniro and Shaham, 2020).

Future directions in Professor David Moorman’s laboratory (University of Massachusetts Amherst, MA) include two main directions. First, a chemogenetic inactivation approach is currently being used to determine the consequences of dmPFC and vmPFC inhibition at a neural circuitry level, for example, efferent projections from the mPFC to the nucleus accumbens. Second, dmPFC and vmPFC activity is being examined in individual neurons to characterize single neuron firing patterns during distinct aspects of motivational behaviors.
